# Supplementary material for: The Effect of Carbohydrate Intake on Strength and Resistance Training Performance: A Systematic Review
Source: Nutrients. 2022 Feb 18;14(4):856. doi: 10.3390/nu14040856 (PMC8878406; doi:10.3390/nu14040856)
Supplement: Supplementary file 1 [file nutrients-14-00856-s001.zip › nutrients-1588576-supplementary.pdf]

Appendix: systematic search strategy

MEDLINE & SPORTDiscus via EBSCOhost

Date: from 1979 (earliest possible in EBSCOhost) until 31th Dec 2021.

#	Query	Limiters/Expanders	Results
S4	S1 AND S2 AND S3	Search modes – Find all my search terms	608
S3	(MH "Muscle Strength") OR (strength OR 1RM OR performance OR failure OR power OR "total work" OR torque OR force OR volume OR repetitions)	Search modes – Find all my search terms	4,299,030
S2	MH "Resistance Training" OR MH "Weight Lift*" OR (isokinetic OR "strength training" OR "strength exercise*" OR "resistance training" OR "resistance exercise*" OR powerlift* OR weightlift* OR "power lift" OR Crossfit)	Search modes – Find all my search terms	69,390
S1	(MH "Carbohydrates") OR ("glycogen depletion" OR "high carbohydrate" OR "low carbohydrate" OR keto* OR (maltodextrin N2 (supplement* OR intake) OR (glucose N2 (ingestion OR intake OR supplement) OR (carbohydrate* N6 (intake* OR supplement* OR manipulat* OR consumption OR ingestion OR feeding OR restricti* OR diet OR drink OR breakfast))	Search modes – Find all my search terms	196,870

SciELO

Date: until 31th Dec 2021.

("glycogen depletion" OR carbohydrate* OR keto* OR maltodextrin OR glucose OR breakfast) AND (isokinetic OR "strength training" OR "resistance training" OR "resistance exercise*" OR powerlift* OR weightlift* OR "power lift" OR Crossfit) AND (strength OR 1RM OR performance OR failure OR power* OR "total work" OR torque OR force OR volume OR repetitions)

Results: 20
